# Natural disasters and renal replacement therapy: lessons from the recent flood in Rio Grande do Sul, Brazil. Anticipation, solidarity, and hope

**DOI:** 10.1590/2175-8239-JBN-2025-L001en

**Published:** 2025-03-14

**Authors:** Roberto C. Manfro, Dirceu Reis da Silva

**Affiliations:** 1 Universidade Federal do Rio Grande do Sul, Faculdade de Medicina, Porto Alegre, RS, Brazil.; 2 Hospital de Clínicas de Porto Alegre, Porto Alegre, RS, Brazil.

The world is experiencing numerous catastrophic weather events that affect entire populations. Consequently, many health services are facing threats to their infrastructure and workforce, affecting their ability to provide quality healthcare. In May 2024, the southern Brazilian state of Rio Grande do Sul (RS), which has a population of around 11 million people, was struck by a major flood, considered the most significant climate catastrophe in its history. The disaster affected 95% of the state’s 478 municipalities, affecting approximately 2.4 million people^1^. Of these, around 600,000 people were displaced and more than 80,000 required shelter^1^. These included individuals with end-stage renal disease requiring dialysis and transplant recipients. In Porto Alegre, the city, entire neighborhoods were flooded and evacuated, causing severe damage to a large hospital and numerous other healthcare facilities that were inundated, while many additional hospitals and facilities were partially affected. This disruption was primarily due to insufficient water supply and the inability of workers to reach these sites. A humanitarian corridor was established to prioritize the transport of patients and health professionals to their or other hospitals and health services. Tragically, 182 people died and dozens are still missing^1^.

People undergoing dialysis therapies and transplant recipients are particularly dependent on support structures to ensure proper treatment. Major natural disasters can disrupt these structures and put their lives in immediate danger. The lack of available dialysis facilities and delivery of immunosuppressive drugs, provided free of charge by the government, along with disrupted hospitals, are perhaps the most critical threats in such situations. In RS, there are approximately 7,500 patients undergoing dialysis treatment and around 6,800 people living with a transplanted organ.

The May floods affected 46 of the 70 renal replacement therapy (RRT) facilities in 29 cities across RS, impacting 4,000 patients^2^. At the onset of the disaster, major road disruptions hindered patients’ ability to get from their municipalities to hemodialysis services. Hemodialysis patients were rerouted to distant more dialysis units, and some peritoneal dialysis patients lost their bath supplies and cyclers. Patients and healthcare professionals in several regions were displaced from their homes, requiring shelter and social support. Hospitals, clinics, patients, the aviation sector, the military, civil defense, and government agencies worked closely together and mobilized resources and supplies to address the crisis.

Beyond the challenge of reaching the dialysis facilities, numerous other issues arose. These included ensuring a water supply for the dialysis units, often by water trucks, maintaining a supply of dialysis electrolyte solutions (two major companies producing these solutions were inoperative for weeks), and securing dialysis filters, lines, and other necessary medications and materials. With stocks usually sufficient for one week, around 30 dialysis facilities experienced shortages of these materials within the first month^2^. To provide treatment to everyone in need, many facilities had to reduce hemodialysis session times and create additional shifts. This led to frequent fluid overloads, which were managed with extra dialysis sessions, and hyperkalemia situations, which were addressed in some major dialysis units with potassium binders donated by the industry.

Patients on peritoneal dialysis, who make up approximately 6% of RRT patients in RS, had to switch from automated to manual procedures due to power outages or the loss of their cyclers, which were damaged or washed away by the floods. Despite these challenges, there were no recorded deaths among dialysis patients due to the unavailability of treatments. Organ transplant activity experienced a significant 40% reduction due to a drastic decrease in the state’s organ procurement capability (source: RS State Transplant Agency). The flooding of the main airport also hindered the ability to receive organs from other states, further compounded by operational restrictions in hospitals. The supply of immunosuppressive medications was maintained through redistribution from unaffected areas, the efforts of non-governmental organizations, and the collaborative efforts among patients.

The response to the May floods in RS underscored the importance of community resilience in providing rapid assistance, including to dialysis and transplant patients. The entire community worked together in a coordinated effort, devising and implementing rescue strategies that saved hundreds of lives. As we face the undeniable impacts of global climate change and become increasingly vulnerable to unprecedented environmental disasters, there is a pressing need for health services to adapt and prepare for these new realities. Effective adaptations will require the active engagement of numerous stakeholders, as well as careful planning, effective communication, collaboration, and solidarity to safeguard health and lives.
